# The Effect of a TLR3 Agonist on Airway Allergic Inflammation and Viral Infection in Immunoproteasome-Deficient Mice

**DOI:** 10.3390/v16091384

**Published:** 2024-08-29

**Authors:** Niccolette Schaunaman, Taylor Nichols, Diana Cervantes, Paige Hartsoe, Deborah A. Ferrington, Hong Wei Chu

**Affiliations:** 1 National Jewish Health, Denver, CO 80206, USA; schaefern@njhealth.org (N.S.); cervantesd@njhealth.org (D.C.); hartsoep@njhealth.org (P.H.); 2 Doheny Eye Institute, University of California Los Angeles, Arcadia, CA 91007, USA; dferrington@doheny.org

**Keywords:** immunoproteasome, rhinovirus, allergic asthma

## Abstract

Allergic asthma is characterized by increased type 2 inflammation, including eosinophils. Subjects with allergic asthma have recurrent symptoms due to their constant exposure to environmental allergens, such as house dust mite (HDM), which can be further exacerbated by respiratory infections like rhinovirus. The immunoproteasome (IP) is a proteolytic machinery that is induced by inflammatory mediators during virus infection, but the role of the IP in airway allergic inflammation during rhinovirus infection remains unknown. Wild-type (WT) and IP knockout (KO) mice were challenged with HDM. At 48 h after the last HDM challenge, mice were infected with rhinovirus 1B (RV-A1B) for 24 h. After HDM and RV-A1B treatment, IP KO (vs. WT) mice had significantly more lung eosinophils and neutrophils, as well as a significantly higher viral load, but less IFN-beta expression, compared to WT mice. A TLR3 agonist polyinosinic-polycytidylic acid (Poly I:C) treatment after RV-A1B infection in HDM-challenged IP KO mice significantly increased IFN-beta expression and reduced viral load, with a minimal effect on the number of inflammatory cells. Our data suggest that immunoproteasome is an important mechanism functioning to prevent excessive inflammation and viral infection in allergen-exposed mice, and that Poly I:C could be therapeutically effective in enhancing the antiviral response and lessening the viral burden in lungs with IP deficiency.

## 1. Introduction

Allergic asthma is the most prevalent phenotype of asthma [1,2,3]. It is associated with enhanced response to inhaled allergens, leading to inflammatory cell infiltration (i.e., eosinophils) into the airways, airway obstruction, and wheezing [2,3]. The onset of allergic asthma is most often in childhood, where it is reported that about 80% of childhood asthma has an allergic component, and can continue into adulthood [4]. Allergic inflammation in conjunction with respiratory infections can lead to persistent asthma [3,5,6]. Upper-respiratory infection with rhinovirus (RV) such as RV-A1B is the most common cause of the common cold and is a contributing factor to asthma exacerbations [7,8,9]. With the lack of effective therapy for RV infections, it is critical to understand the mechanisms of viral exacerbations of allergic asthma.

The immunoproteasome (IP) is an inducible version of the constitutive proteasome [10,11,12,13,14]. After pro-inflammatory stimulation from interferons (IFNs) or TNFα, the three proteolytic subunits of the constitutive proteasome (β1, β2, and β5) are replaced by LMP2, MECL-1, and LMP7 inducible subunits, which assemble into the IP [15,16,17]. The IP has been studied during adaptive immunity, as it has functions in cleaving peptides for antigen presentation [17,18,19,20]. IP deficiency has been described in lung cancer as a mechanism of immune evasion of cancer cells [21]. The role of the IP in host immune response to infections and its underlying mechanisms remain poorly understood. The limited amount of work in the literature suggests that the IP may process viral epitopes functioning to promote CD8+ T cell activation as an antiviral mechanism [22]. On the other hand, viruses evade the antiviral response by blocking IP formation induced by IFNs. So far, the role of the IP in host defense against viral infection in allergic asthma remains unclear. Our group has recently studied the role of the IP in innate immune response to RV infections [23,24]. We found that IP knockout (KO) mice (specifically, LMP2 and LMP7 subunit KO) infected with RV-A1B had significantly higher neutrophils in bronchoalveolar lavage (BAL) fluid, as well as increased viral load [23]. We also found that treating mice with a low dose of polyinosinic:polycytidylic acid (Poly I:C), a double-stranded viral mimic, before infection was able to prevent excessive neutrophilic inflammation in IP KO mice. While we found that the IP is important in inhibiting RV-induced inflammation, our previous work did not delve into the role of the IP in an allergic asthma setting, with or without viral infection.

In this report we hypothesized that IP deficiency exaggerates allergen-induced inflammation following virus infection and increases viral load by inhibiting antiviral gene expression. As a TLR3 agonist, Poly I:C has been shown to enhance interferon responses during viral infection [25,26]; we further hypothesized that giving Poly I:C after the infection serves as a therapeutic approach working to reduce the severity of respiratory viral infection in IP KO mice.

## 2. Materials and Methods

### 2.1. Rhinovirus 1B (RV-A1B) Preparation

RV-A1B (American Type Culture Collection (ATCC), Manassas, VA, USA) were propagated in H1-Hela cells (CRL-1958, ATCC), purified, and titrated to plaque-forming unit (PFU), as described previously [23,24,27].

### 2.2. Mice

Wild-type (WT) C57BL/6 mice and immunoproteasome (IP) knockout (KO) mice, either LMP2 or LMP7 subunit KO, on a C57BL/6 background, were kindly provided by Dr. Deborah A. Ferrington of the Doheny Eye Institute, Pasadena, CA, USA [28,29]. All mice were bred at the National Jewish Health (NJH) biological resource center (BRC) under pathogen-free housing conditions. All of the experimental protocols were approved by the Institutional Animal Care and Use Committee at NJH.

### 2.3. Mouse Model of House Dust Mite (HDM) Challenge and Viral Infection

While the LMP2 and LMP7 subunits have different peptidase activity in the immunoproteasome [15], in our model, LMP2 and LMP7 KO mice did not show any statistically significant differences from each other in any assay, including specifically the HDM+RV-A1B condition, and as such, we combined them into one IP KO group.

To induce mouse allergic airway-inflammation, WT and IP KO mice (n = 5–17 mice per group, 8–12 weeks of age, and gender-matched) were intranasally sensitized with 10 µg/mouse of HDM (Greer laboratories, Lenoir, NC, USA) or 50 µL of phosphate-buffered saline (PBS, pH 7.0) on days 0 and 7. Mice were then challenged once a day for three consecutive days (days 14–16) with 10 µg of HDM or 50 µL PBS [30,31]. At 48 h after the last HDM challenge, mice were infected with 5 × 10^6^ PFU/mouse of RV-A1B or 50 µL of PBS (pH 7.0) [23,24,32,33]. At 24 h post-infection, the mice were sacrificed. We chose this time point based on our previous publication utilizing our rhinovirus infection model in LMP2 whole body KO mice [24]. Lungs were lavaged with 1ml of sterile saline. Cell-free BAL fluid was used for viral load measurement and cytokine measurement. BAL fluid cell-differential cytospin slides were stained with a Diff-Quick stain kit (IMEB, San Marcos, CA, USA) for cell differential counts. Leukocyte differentials were determined as a percentage of 500 counted leukocytes. Half of the left lung was used for viral load and interferon mRNA expression. Lung tissue was fixed in 10% formalin and embedded in paraffin for lung histopathology using H/E staining.

### 2.4. Poly I:C Treatment in Mice with HDM Challenge and RV-A1B Infection

To determine whether Poly I:C acts as a therapeutic, after two hours post-infection, HDM-challenged and RV-A1B-infected IP KO mice (n = 7–13 mice per group) were given either 5 µg/mouse of Poly I:C (Invivogen, San Diego, CA, USA) or 50 µL PBS (pH 7.0) via intranasal inoculation. At 24 h post-infection, mice were sacrificed for viral load, interferon mRNA, and cytokine measurements, as well as leukocyte counts.

### 2.5. ELISAs

Eotaxin-2 (CCL24) was measured using DuoSet ELISA kits (R&D Systems, Minneapolis, MN, USA) according to the manufacturer’s specifications.

### 2.6. Reverse Transcription and Quantitative Real-Time PCR (RT-qPCR)

RNA was extracted from BAL and homogenized lung tissue, as described previously [24,34]. Briefly, equal volumes of BAL fluid were used to extract RNA using Mini Spin Columns (Epoch Life Science Inc., Missouri City, TX, USA) according to the manufacturer’s instructions. RV-A1B and interferons were measured from homogenized lung tissue taken from half of the left lung, and RNA was isolated using the TRIzol reagent method. RNA was then reversely transcribed to cDNA.

Custom primers and probes (Integrated DNA Technologies, Coralville, IA, USA) were designed for RV-A1B and Interferon (IFN)-beta. The specific primers and probes used to amplify RV-A1B were 5′-CCTCCGGCCCCTGAAT-3′ (forward primer); 5′-GGTCCCATCCCGCAATT-3′ (reverse primer); and 5′-CTAACCTTAAACCTGCAGCCA-3′ (probe) [23]. The specific primers and probes used to amplify IFN-beta were 5′-GACGGAGAAGATGCAGAAGAG-3′ (forward primer); 5′-CCACCCAGTGCTGGAGAA-3′ (reverse primer); and 5′-TGCCTTTGCCATCCAAGAGAT-3′ (probe) [31]. Target gene expression was normalized to the housekeeping gene 18S rRNA (ThermoFisher, Waltham, MA, USA). The comparative threshold cycle method (∆∆Ct) was applied to determine the relative levels of IFN-beta using the RV-A1B-infected WT mice for the HDM-challenge mouse model, and the PBS treated IP KO mice in the Poly I:C model as the controls.

Viral load in cell-free BAL fluid was assessed by extracting total RNA from the same volume (i.e., 50 µL) of BAL fluid in all mice, synthesizing cDNA, and performing qPCR using the primers and probe detailed above.

To quantify viral load in homogenized lung tissue and BAL fluid, RNA was extracted from a stock of RV-A1B with known PFU, cDNA was synthesized and 10-fold serially diluted, and then qPCR was performed to generate a standard curve. PFU equivalents were calculated from the standard curve using either the cycle threshold (Ct) values from homogenized lung tissue, or the Ct values from the BAL fluid.

### 2.7. Statistical Analysis

GraphPad PRISM version 10.0 software was used for all statistical analysis. Two group comparisons of nonparametric data were performed using the Mann–Whitney test. A *p*-value < 0.05 was considered statistically significant.

## 3. Results

### 3.1. IP Deficiency Exacerbates Allergic Airway Inflammation after RV-A1B Infection

LMP2 and LMP7 KO mice were combined into a single group, as there were no statistical differences between these strains under HDM+RV-A1B conditions for all outcomes except neutrophil numbers.

**Figure 1:**
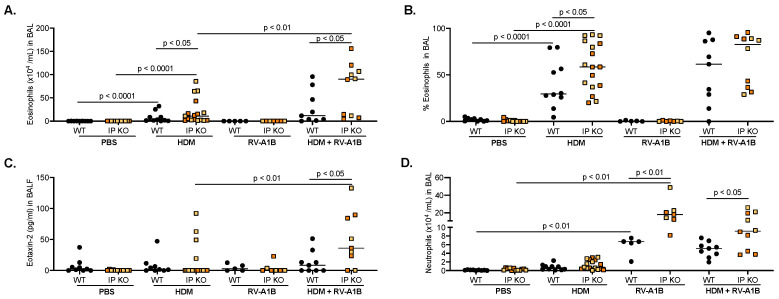
Immunoproteasome (IP) deficiency promotes excessive inflammation following allergen challenge and virus infection. Wild-type (WT) and IP knockout (KO) mice were challenged with house dust mite (HDM) and then infected with RV-A1B for 24 h. IP KO mice had more eosinophils (as to both number (**A**) and percentage (**B**)) and eotaxin-2 (**C**), compared to WT mice after HDM challenge and viral infection. Neutrophil levels (**D**) were significantly higher in RV-A1B-infected IP KO mice in the absence or presence of HDM challenge. N = 5–17 mice per group. Yellow squares indicate individual LMP7 KO mice, and orange squares are individual LMP2 KO mice. Data were analyzed using the Mann–Whitney test.

After HDM challenge, there was a significant increase in eosinophils in both strains of mice (Figure 1A) compared to PBS controls, with IP KO mice having significantly more eosinophils than the WT mice. IP KO, but not WT, mice treated with HDM and RV-A1B had significantly more eosinophils than their HDM-alone counterparts. Importantly, HDM-challenged and RV-A1B-infected IP KO mice maintained a significantly higher level of eosinophils compared to WT mice. These trends were also seen in eosinophil percentage (Figure 1B). Similarly, eotaxin-2, an eosinophil chemokine, had significantly higher levels in IP KO mice after HDM and RV-A1B infection than were found in WT mice (Figure 1C). Neutrophils were significantly upregulated in both strains of mice (Figure 1D) following RV-A1B infection, with IP KO mice having significantly more than WT mice. Neutrophil numbers were similar to those for RV-A1B alone when mice were treated with both HDM and RV-A1B. H/E-stained lung tissue slides confirmed the infiltration of eosinophils, neutrophils, and other types of inflammatory cells in the lungs of IP KO and WT mice (Supplementary Figure S1). Our data suggest that an IP deficiency led to an excessive inflammatory response initiated by HDM and RV-A1B.

### 3.2. IP Deficiency Increases Viral Load and Inhibits Antiviral Gene Expression

**Figure 2:**
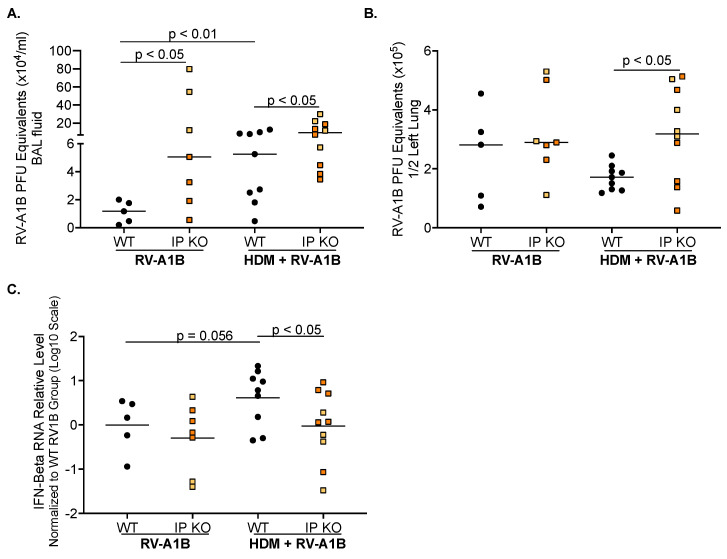
IP deficiency increases viral load but fails to elicit an antiviral response after house dust mite (HDM) challenge and virus infection. Higher viral loads were present in the BAL fluid (**A**) and lung tissue (**B**) of IP KO mice, compared to WT mice, after HDM and RV-A1B infection. There was less interferon-beta mRNA expression (**C**) in IP KO mice, compared to WT mice. N = 5–17 mice per group. Yellow squares indicate individual LMP7 KO mice, and orange squares are individual LMP2 KO mice. Data were analyzed using the Mann–Whitney test.

When RV-A1B was measured in BAL fluid (Figure 2A), IP KO mice had significantly more virus present compared to WT mice. With the addition of HDM, WT mice had an increase in viral release compared to RV-A1B infection alone. Interestingly, HDM did not further increase viral load released into the BAL fluid of IP KO mice, but IP KO mice continued to have significantly higher viral load than WT mice. Figure 2B shows similar viral loads in homogenized lung tissue between RV-A1B-infected IP KO and WT mice without HDM treatment. When mice were given both HDM and RV-A1B, IP KO mice showed significantly more viral load in homogenized lung tissue than the WT mice. When IFN-beta mRNA expression was measured (Figure 2C), there was no difference between RV-A1B-infected WT and IP KO mice without HDM treatment. With the addition of HDM, WT mice trended to have more IFN-beta mRNA expression compared to RV-A1B infection alone, while HDM-challenged IP KO mice had similar IFN-beta levels compared to their RV-A1B-infected counterparts. It is important to note that IP KO mice had significantly less IFN-beta mRNA expression than WT mice after HDM treatment and RV-A1B infection. Our data suggest that in allergic airways, IP deficiency was not able to elicit a proper antiviral response.

### 3.3. Poly I:C Increases IFN-Beta Expression and Decreases Viral Load in IP KO Mice Treated with HDM

**Figure 3:**
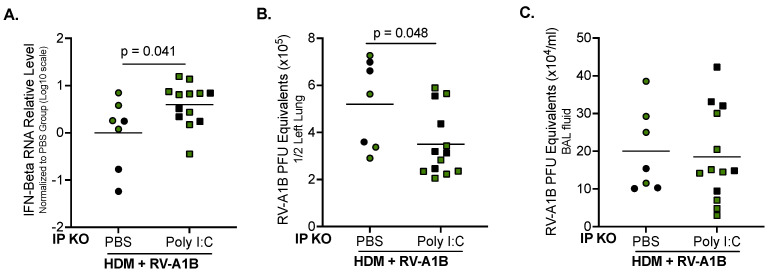
Poly I:C treatment in IP-deficient mice rescues the antiviral response and reduces the viral load. Poly I:C (5 μg/mouse) given to IP KO mice after HDM and RV-A1B infection significantly enhanced IFN-beta mRNA expression (**A**) and decreased RV-A1B load in homogenized lung tissue (**B**); Poly I:C did not change viral load (**C**) in BAL fluid (BALF). N = 7–13 mice per group. Black symbols ae LMP7 KO mice, and green symbols are LMP2 KO mice. Data were analyzed using the Mann-Whitney test.

When HDM-challenged IP KO mice were given 5 µg of Poly I:C two hours after RV-A1B infection, they showed a significant increase in IFN-beta mRNA expression compared to their counterparts with PBS treatment (Figure 3A). Poly I:C treatment also significantly reduced viral load in the lung tissue (Figure 3B). Interestingly, Poly I:C did not change the level of virus released into the BAL fluid of the IP KO mice (Figure 3C).

**Figure 4:**
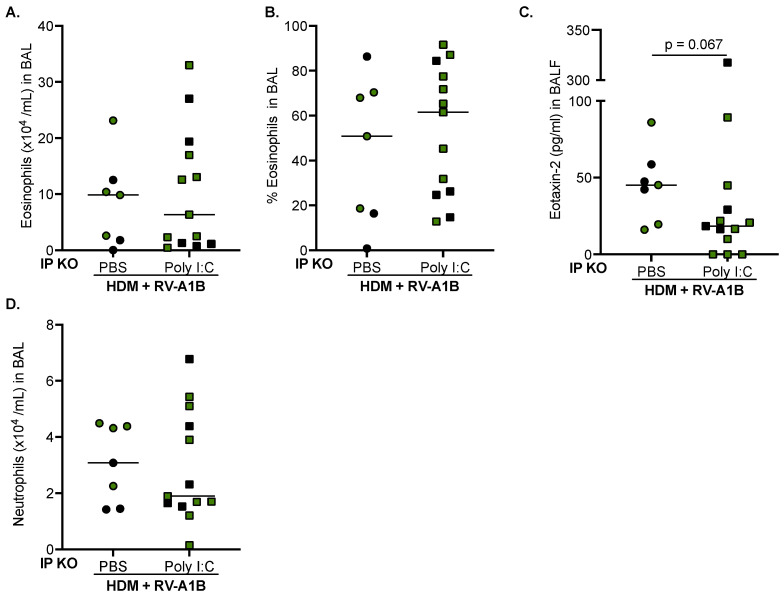
Poly I:C treatment in IP-deficient mice does not increase airway inflammation. Poly I:C (5 μg/mouse) given to IP KO mice after HDM and RV-A1B infection did not change the number (**A**) or percentage (**B**) of eosinophils, eotaxin-2 levels (**C**), or neutrophils (**D**) found in the BAL fluid (BALF). N = 7–13 mice per group. Black symbols are LMP7 KO mice, and green symbols are LMP2 KO mice. Data were analyzed using the Mann–Whitney test.

While the numbers and percentages of eosinophils remained similar (Figure 4A,B), there was a trend of decreased eotaxin-2 levels in the BAL fluid of IP KO mice given Poly I:C (Figure 4C). Importantly, neutrophil numbers remained unchanged (Figure 4D). Our data suggest that the dose of Poly I:C treatment we used can enhance the antiviral response in IP KO mice without worsening airway inflammation.

## 4. Discussion

We have shown for the first time that the immunoproteasome is important in inhibiting excessive allergic inflammation and viral infection. We have also shown that Poly I:C may promote an antiviral response and reduce viral load in IP-deficient lungs.

We are aware of the controversy about the role of the IP in airway allergic-inflammation. Oliveri et al. found that treatment with an immunoproteasome inhibitor in mice challenged with ovalbumin (OVA) or HDM significantly reduced IL-4-expressing CD4+ T cells in the lung compared to the vehicle treatment. However, IgE in the serum was not changed when comparing mice treated with and without the inhibitor [35]. Similarly, Volkov et al. found that LMP7 KO mice given aerosolized OVA showed a reduction in eosinophils compared to WT mice; however, when challenged with HDM, eosinophil numbers were similar between WT and LMP7 KO mice [36]. In contrast to the major findings of the other groups, Chen et al. found that LMP7-deficient macrophages showed an increase in IL-4-induced M2 polarization [37]. Our data in HDM-challenged mice without viral infection support a role of the IP in suppressing eosinophilic inflammation.

IP assembly has been studied extensively for years, and many groups have shown that there are different roles for each of the subunits during assembly. LMP2 is necessary for MECL-1 to be incorporated, and LMP7 is necessary for the maturation of the immunoproteasome [22,38,39]. LMP7-deficient mice have been shown to have less LMP2 and MECL-1 subunit expression [13]. The lack of LMP2 or LMP7 similarly reduced numbers of CD8+ cells or was associated with a reduction in MHC class I antigen presentation [29,40,41,42]. How or if the assembly of the immunoproteasome is dysregulated in our individual IP-subunit-deficient mice was not investigated, but utilizing a triple-deficient mouse model in the future could help determine the role of the immunoproteasome under an allergic setting, as opposed to looking at the individual subunits.

While respiratory viruses such as rhinovirus have been linked to asthma-like symptoms [43,44,45], much of the literature is conflicting on the question of whether virus infection, combined with allergen challenge, leads to enhanced allergic inflammation. Mehta and Croft found that after HDM and RV-A1B infection, mice had significantly more eosinophils than after HDM treatment alone [46]. Similarly, Siegle et al. attempted to mimic an early-in-life infection followed by OVA challenge. They found that following pneumonia virus of mice (PVM) and OVA challenge, mice had significantly more intraepithelial eosinophils compared to PVM alone [47]. Conversely, Looi et al. found that mice given either OVA or HDM followed by influenza infection had similar levels of eosinophils compared to OVA or HDM alone [43]. The disparity in these reports could be due to the differences in allergens, the different types of viruses, or the timing and doses of these stimuli given together. Our data suggest that under non-allergic conditions, RV infection does not enhance eosinophilic inflammation. However, our work does suggest that the IP is important in allergic inflammation regulation in the presence of viral infection. While the exact mechanism of how the IP is inhibiting eosinophilic inflammation needs further study, there are studies suggesting that the IP may be able to regulate IL-4 signaling [37].

As we have previously found that IP KO mice have significantly higher viral loads than WT mice [23,24], we wanted to explore whether allergen challenge worsened viral infection. Compared to WT mice, viral loads for both lung tissue and BAL were significantly higher in IP KO mice after allergen challenge, which was coupled with less IFN-beta mRNA expression. This suggests that after RV-A1B infection, IP KO mice are unable to elicit a proper antiviral response in the presence of HDM, leading to increased viral load. Uller et al. found that bronchial epithelial cells from asthma patients exposed to a viral mimic had significantly less IFN-beta but more type 2 cytokine thymic stromal lymphopoietin (TSLP) expression than cells isolated from healthy subjects [48]. Our findings could suggest that during allergic asthma, IP-deficient subjects could be more susceptible to viral infections due to the lack of an antiviral response.

Since IP KO mice cannot elicit a proper antiviral response following HDM and RV-A1B infection, we determined whether therapeutic intervention via TLR3 activation was effective. We previously administered Poly I:C before viral infection as a preventive approach in LMP7 KO mice to reduce neutrophilic inflammation and CXCL10 release [23]. The findings from our current study provide evidence that Poly I:C given after viral infection may serve as a therapeutic in IP-deficient mice with allergic asthma, a question which has not been investigated before. Poly I:C treatment after viral infection significantly increased IFN-beta and decreased lung-tissue viral load. Type 1 interferons are known to protect against viral infections [49,50,51,52], leading to IFN-stimulated genes, a process which functions to block viral replication. Importantly, while Poly I:C trended to decrease eosinophils and eotaxin-2 levels, neutrophil levels were not increased by Poly I:C, indicating that the dose of Poly I:C we used is indeed therapeutically effective, as it did not elicit an excessive inflammatory response. High doses of Poly I:C have been shown to induce cytokine release and neutrophilic inflammation [53,54,55], leading to airway remodeling and impaired lung function. Although the exact mechanisms by which the IP regulated the antiviral response in allergen-challenged mice with Poly I:C stimulation have not been explored, the IP has been shown to be involved in metabolism [56], which is associated with RV infection [57]. Our future studies will define new antiviral mechanisms such as metabolic dysfunction due to IP deficiency in viral infection and determine how Poly I:C might restore the metabolic dysfunction to reduce viral infection.

Allergen challenge did not further enhance RV-induced neutrophilic inflammation for either strain of mice in our study. However, IP KO mice had significantly more neutrophils present than WT mice. We have previously found that IP KO mice infected with RV-A1B had significantly higher neutrophilic inflammation compared to WT mice [23,24], a determination which we were able to replicate in this study. Data as to neutrophilic inflammation during allergen challenge and virus infection is controversial, depending on type, dose, and timing of stimulations. One group found that RV-A1B infection after HDM challenge slightly increased neutrophil numbers, but the difference was not significant compared to RV-A1B infection alone. The same group changed the timing and frequency of RV-A1B infection after HDM challenge and found that there was a significant increase in neutrophils compared to RV-A1B alone [46]. Phan et al., using the same dose of HDM as the group above, but as given for 10 consecutive days, and followed by RV-A1B infection, significantly increased RV-A1B-induced neutrophilic inflammation compared to RV infection alone [58]. Interestingly, in a different study that used influenza virus after HDM challenge, there was no change in neutrophil percentage, and when the allergen was changed to OVA, influenza virus-infected mice showed a decrease in neutrophil percentage [47]. In our study, the inhibitory role of the IP on neutrophilic inflammation was not dependent on HDM challenges.

There are several limitations to this study. While we have shown IP KO mice have significantly more inflammation and viral load after allergen challenge, we did not, however, extensively examine the cytokine network (e.g., type 2, type 1, and type 17 cytokines) by means of which the IP may regulate allergic asthma. Second, the models shown here are all acute models of allergen challenge and viral infection. To determine the long-term effects of IP deficiency a more chronic model of allergen exposure and viral infection needs to be developed. Lastly, while LMP2 and LMP7 KO mice did not show statistical differences in any outcomes associated with type 2 inflammation (under HDM+RV-A1B conditions), there was statistical significance in the neutrophil numbers. Future studies should be performed to determine the biological differences in these mice which might impact virus-induced neutrophilic inflammation under allergen challenge.

By utilizing our established allergen challenge model in conjunction with RV infection, we have demonstrated for the first time that IP deficiency enhances airway allergic inflammation and viral infection. Appropriate activation of host defense by a TLR3 agonist could be a therapeutic option for those with IP deficiency who may be more susceptible to allergic asthma worsened by rhinovirus infection.

## Data Availability

The data presented in this study are available within the article.

## Supplementary Materials

The following supporting information can be downloaded at: https://www.mdpi.com/article/10.3390/v16091384/s1, Supplementary Figure S1: Lung histopathology from PBS-treated WT (A) and IP KO mice (B), HDM-treated WT (C) and IP KO mice (D), RV-A1B-infected WT (E) and IP KO mice (F), and HDM + RV-A1B treated WT (G) and IP KO mice (H). Eosinophils are represented by red arrows. Original magnification 200×, higher magnification 400×.
