# Supplementary material for: The Effect of a TLR3 Agonist on Airway Allergic Inflammation and Viral Infection in Immunoproteasome-Deficient Mice
Source: Viruses. 2024 Aug 29;16(9):1384. doi: 10.3390/v16091384 (PMC11437510; doi:10.3390/v16091384)
Supplement: Supplementary file 1 [file viruses-16-01384-s001.zip › viruses-3087109-supplementary.pptx]

## Slide 1
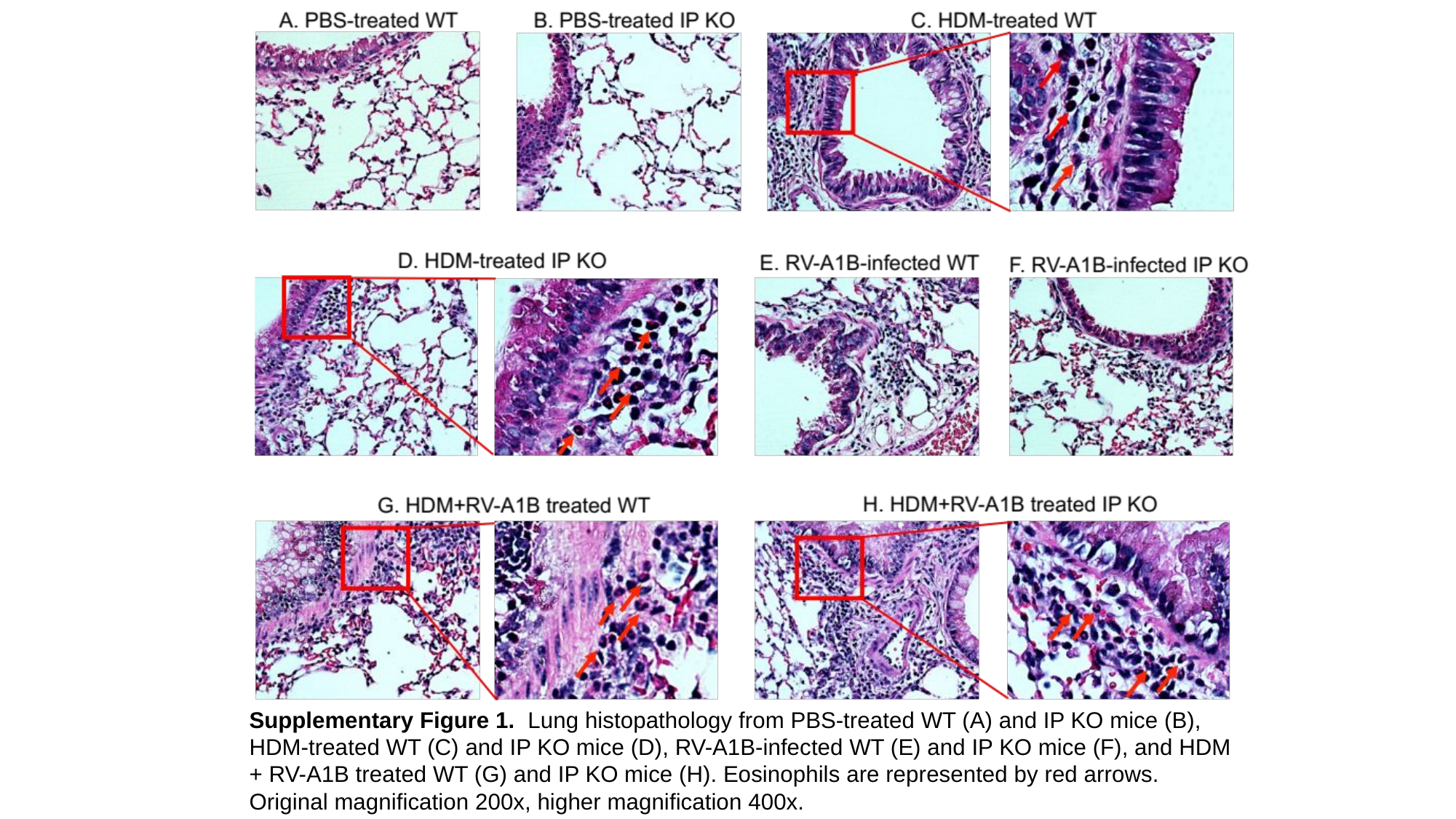

Supplementary Figure 1. Lung histopathology from PBS-treated WT (A) and IP KO mice (B), HDM-treated WT (C) and IP KO mice (D), RV-A1B-infected WT (E) and IP KO mice (F), and HDM + RV-A1B treated WT (G) and IP KO mice (H). Eosinophils are represented by red arrows. Original magnification 200x, higher magnification 400x.
